# Anti-CCP antibodies and bone

**DOI:** 10.1186/s13075-018-1566-3

**Published:** 2018-04-10

**Authors:** Giovanni Orsolini, Ombretta Viapiana, Maurizio Rossini, Giovanni Adami, Cristian Caimmi, Angelo Fassio, Davide Gatti

**Affiliations:** Policlinico G.B. Rossi, Rheumatology Unit, Department of Medicine, Piazzale L. Scuro 10, 37134 Verona, Italy

Dear Editor.

We read with great interest the paper of Cheng et al. [1]. They demonstrate for the first time as anti-cyclic citrullinated peptides antibodies (Anti-CCP) are an independent risk factor for fracture and this data is of high clinical relevance in stratifying rheumatoid arthritis (RA) patients. The authors have a wide population and were able to take into consideration a lot of factors involved in bone disease related and unrelated to RA. They did confirm our previous reports of a negative effect of Anti-CCP on BMD [2], and also showed a relation with FRAX 10 year calculated fracture risk. In particular, the BMD difference was observed at femoral neck, site of cortical bone that is the same of periarticular bone were erosions occur [3, 4]. Nevertheless, the Authors failed to find any correlation between Anti-CCP titer and BMD. This last data is in contrast with other previous clinical reports of a titer dependent effect of Anti-CCP on bone [2, 5].

The reasons of these discrepancies are not straightforward. A possible explanation of lack of correlation between Anti-CCP titer and BMD could be the use of BMD as g/cm^2^ and not as Z-score in the analysis. Z-score has the advantage to evaluate better the bone loss correcting for age and gender. Another possible confounder is the evaluation of glucocorticoids (GCs) as a dichotomous variable. It is not clear how the patients were considered “users” and there were no data on mean dose or duration of use, thus of a cumulative dose. The Authors report also data on anti-osteoporosis medications, but they are unspecified, even if they probably refer to antiresorptive drugs. Moreover, data on vitamin D and calcium, either supplements and serum levels, are completely lacking. Vitamin D and PTH are key determinants of cortical bone (e.g. femoral neck) mass [3], so their inclusion in the analysis would have been of critical importance.

We think the paper is of great interest but that some of the variables taken into consideration should be implemented and that a more complex statistical analysis is needed to take the best from its data.

## Abbreviations

Anti-CCP: anti cyclic citrulline peptides antibodies
BMD: bone mineral density
GCs: glucocorticoids
PTH: parathormone
